# MicroRNA-130a associates with ribosomal protein L11 to suppress c-Myc expression in response to UV irradiation

**DOI:** 10.18632/oncotarget.2728

**Published:** 2014-12-31

**Authors:** Yuhuang Li, Kishore B. Challagundla, Xiao-Xin Sun, Qinghong Zhang, Mu-Shui Dai

**Affiliations:** ^1^ Departments of Molecular & Medical Genetics, School of Medicine and the OHSU Knight Cancer Institute, Oregon Health & Science University, Portland, OR 97239, USA; ^2^ Department of Dermatology, University of Colorado, Denver, Aurora, CO 80045, USA

**Keywords:** miR-130a, L11, c-Myc, microRNA, UV irradiation

## Abstract

The oncoprotein c-Myc is essential for cell growth and proliferation while its deregulated overexpression is associated with most human cancers. Thus tightly regulated levels and activity of c-Myc are critical for maintaining normal cell homeostasis. c-Myc is down-regulated in response to several types of stress, including UV-induced DNA damage. Yet, mechanism underlying UV-induced c-Myc reduction is not completely understood. Here we report that L11 promotes miR-130a targeting of *c-myc* mRNA to repress c-Myc expression in response to UV irradiation. miR-130a targets the 3′-untranslated region (UTR) of *c-myc* mRNA. Overexpression of miR-130a promotes the Ago2 binding to *c-myc* mRNA, significantly reduces the levels of both c-Myc protein and mRNA and inhibits cell proliferation. UV treatment markedly promotes the binding of L11 to miR-130a, *c-myc* mRNA as well as Ago2 in cells. Inhibiting miR-130a significantly suppresses UV-mediated c-Myc reduction. We further show that L11 is relocalized from the nucleolus to the cytoplasm where it associates with *c-myc* mRNA upon UV treatment. Together, these results reveal a novel mechanism underlying c-Myc down-regulation in response to UV-mediated DNA damage, wherein L11 promotes miR-130a-loaded miRISC to target *c-myc* mRNA.

## INTRODUCTION

The c-Myc oncoprotein is essential for normal cell growth and proliferation by regulating the expression of a large number of genes involved in cell cycle, apoptosis, differentiation, angiogenesis, metabolism, ribosomal biogenesis, and stem cell renewal [1–3]. However, deregulated overexpression and activation of c-Myc contribute to a broad range of human cancers [4]. Thus, c-Myc level and activity must be tightly regulated during normal homeostasis and turning down c-Myc level and activity in cancer cells has therapeutic significance.

In normal cells, c-Myc is tightly regulated at multiple levels [3] and these mechanisms can be disrupted in cancer cells. c-Myc transcription is transiently activated by growth factor and mitogenic stimuli and controlled by multiple promoter elements at the *c-myc* gene [3, 5, 6]. c-Myc translation can be regulated at both the 5′-untranslated region (UTR) and the 3′-UTR [7, 8]. c-Myc protein stability is subjected to a multitude of tight posttranslational regulation via the ubiquitin-dependent proteasome system [9–11]. Likewise, *c-myc mRNA* stability is regulated by a translation-independent mechanism involving an AU-rich element (ARE) at its 3′-UTR [12, 13] and a translation-dependent mechanism involving an ~250 nucleotide (nt) coding region instability determinant (CRD) [14, 15]. Several ARE binding proteins, including AUF1 [16], HuR [17], and tristetraprolin (TPP) [18] have been found to bind *c-myc* ARE and act as *c-myc* mRNA destabilizing factors. CRD binding protein (CRD-BP) binds to the CRD, leading to the protection of *c-myc* mRNA from endoribonuclease cleavage within CRD [14, 15]. Finally, *c-myc mRNA* stability and/or translation are negatively regulated by several microRNAs (miRNAs), such as Let-7 [19], miR-145 [20], miR-34c [21], miR-24 [22, 23], and miR-185 [24]. Together, c-Myc is precisely regulated to coordinate with normal cell growth and proliferation.

c-Myc also needs to be tightly controlled under stress conditions. To overcome cellular stress and maintain genomic integrity, cells develop mechanisms to slow down cell cycle progression allowing cells to recover from the damage or eliminate the cells from the replicating pool if the damage is irreparable. One of the key mechanisms is p53-dependent cell cycle checkpoint that is activated by almost all kinds of stress, including DNA damage such as ultraviolet (UV) and γ-irradiation, oncogenic and ribosomal stress [25–27]. It has been shown that c-Myc overactivation can induce genomic instability [3, 28]. Thus, c-Myc needs to be tightly controlled in order to coordinate with stalled cell cycle progression in response to stress. Indeed, c-Myc protein is reduced by treatment of cells with UV irradiation [29] and other DNA damaging agents [30]. However, the mechanisms underlying the c-Myc down-regulation in response to DNA damage are not completely understood.

We previously found that ribosomal protein L11 (L11 thereafter) regulates c-Myc levels via miR-24-mediated *c-myc* mRNA decay in response to ribosomal stress [22]. miRNAs are a class of small endogenous non-coding RNAs controlling the activity of ~50% of all protein-coding genes in mammals (33). Mature miRNAs are single stranded RNAs of ~23 nt in length that negatively regulate gene expression by base pairing to partially or perfectly complementary sites on the target mRNA, usually in the 3′-UTR, to affect the translation and/or mRNA stability [31–33]. miRNAs play key roles in the regulation of diverse cellular processes [31]; deregulation of miRNAs is associated with the development of various human diseases including cancers [34–36]. L11 was initially found to be essential for p53 activation in response to ribosomal stress induced by perturbation of ribosomal biogenesis [37–39]. Ribosomal stress is often accompanied by the disruption of the nucleolus, leading to the relocation of the nucleolar components including ribosomal proteins into the nucleoplasm [40, 41]. Intriguingly, disruption of the nucleolus is also a common event in cells following DNA damage including UV irradiation [42], suggesting that L11 may play a role in regulating c-Myc via miRISC in response to DNA damage as well.

In this study, we found that L11 recruits miR-130a-3p (miR-130a thereafter) to target *c-myc* mRNA following UV irradiation. Overexpression of miR-130a decreases both *c-myc* mRNA and protein and inhibits cell proliferation. UV damage induces the release of L11 from the nucleolus to the cytoplasm where it recruits miR-130a-associated RNA interference silencing complex (miRISC) to target *c-myc* mRNA at its 3′-UTR. Thus our results uncover a novel function of miR-130a in suppressing c-Myc in response to DNA damage.

## RESULTS

### L11 associates with miR-130a

**Figure 1:**
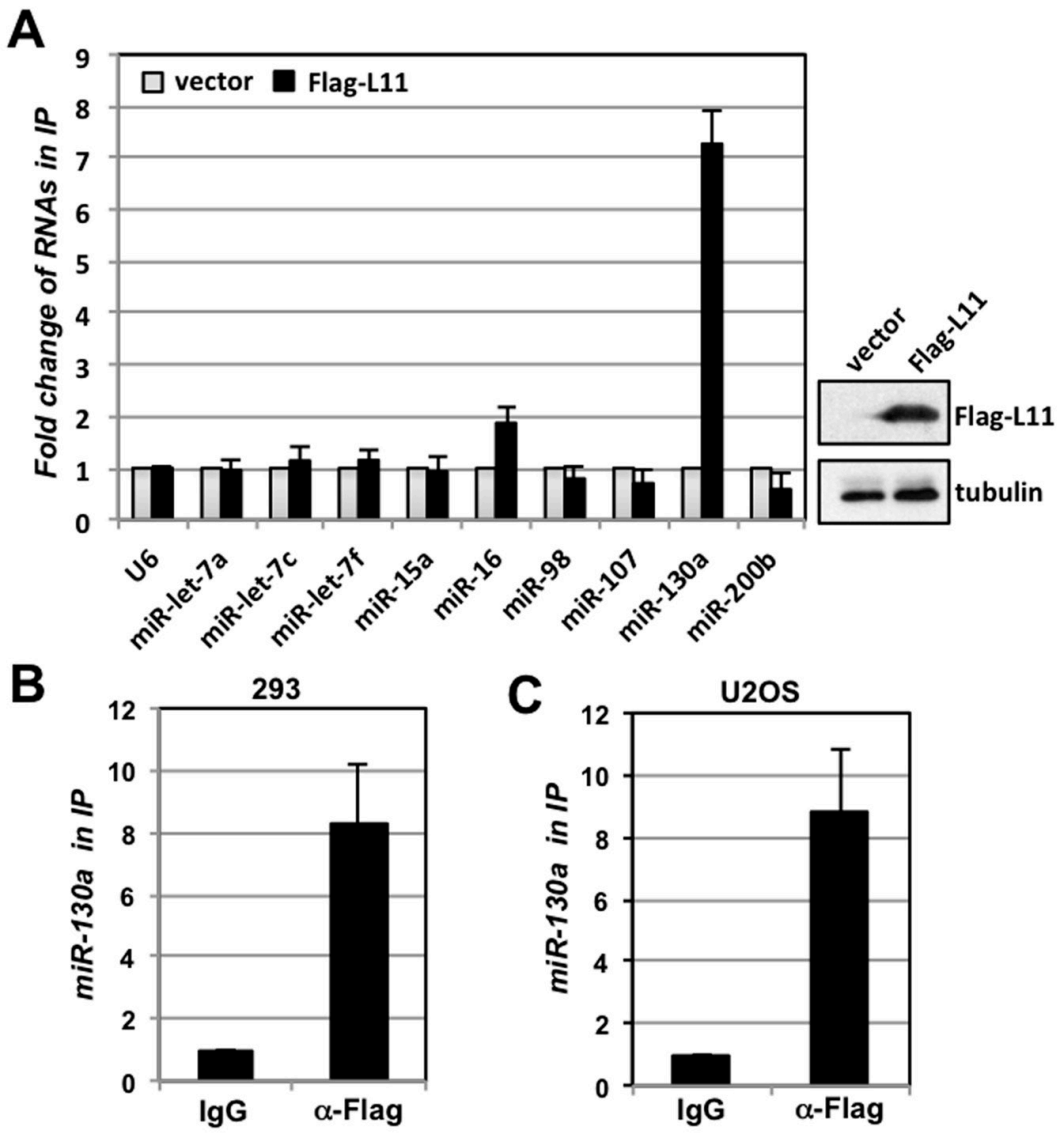
L11 associates with miR-130a **(A)** Identification of miR-130a as a L11-associated miRNA. 293 cells transfected with control or Flag-L11 were subjected to RNA-IP using anti-Flag antibody followed by detection of indicated miRNAs using RT-qPCR. The expression of Flag-L11 is shown in the right panel. **(B–C)** L11 associates with miR-130a in cells. Lysates from 293 (B) and U2OS (C) cells transfected with Flag-L11 were immunoprecipitated with control mouse IgG or anti-Flag antibody, followed by RT-qPCR detection of miR-130a.

We have previously shown that L11 associates with miR-24, but not other Myc-targeting miRNAs including let-7b and miR-34c, to repress c-Myc expression in response to ribosomal stress [22]. To further elucidate the role of L11 in the regulation of c-Myc, we sought to examine whether it could associate with other miRNAs that negatively regulate cell growth and proliferation. We performed RNA-IP assays with anti-Flag antibody using lysates from 293 cells transfected with control or Flag-L11 plasmid. RNAs extracted from the immunoprecipitates were assayed by RT-qPCR for a panel of miRNAs with potential tumor suppressor function, including miR-15a, miR-16, miR-130a, miR-107, miR-200b, and several let-7 family members including let-7a, let-7c, let-7f and miR-98 [35, 43–48]. As shown in Fig. 1A, L11 bound strongly to miR-130a and to a less extent to miR-16, but not other tested miRNAs. To verify this L11-miR-130a association, we performed similar RNA-IP experiments in cells transfected with Flag-L11 using IgG control. Indeed, miR-130a was specifically immunoprecipitated by anti-Flag antibody, but not control IgG, in both 293 (Fig. 1B) and U2OS (Fig. 1C) cells, suggesting that L11 associates with miR-130a in cells.

### miR-130a regulates c-Myc levels

**Figure 2:**
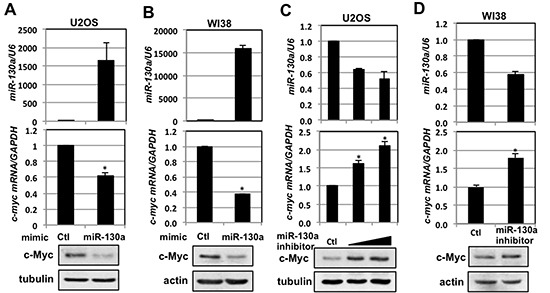
miR-130a regulates c-Myc levels **(A–B)** Overexpression of miR-130a decreases c-Myc levels. U2OS (A) or WI38 (B) cells transfected with control or miR-130a mimics were assayed for the relative expression of miR-130a normalized with U6 snRNA (top panels), *c-myc* mRNA normalized with *GAPDH* mRNA (middle panels) by RT-qPCR, and c-Myc protein levels (bottom panels) by IB. *p < 0.01, compared with control transfected cells. **(C–D)** Inhibition of miR-130a increases c-Myc levels. U2OS (C) or WI38 (D) cells transfected with control or miR-130a hairpin inhibitors were assayed for the relative expression of *c-myc* mRNA normalized with *GAPDH* mRNA (middle panels) by RT-qPCR and c-Myc protein expression (bottom panels) by IB. *p < 0.01, compared with control transfected cells.

miR-130a has recently been shown to suppress cancer cell growth and invasion through targeting the proto-oncogene MET [43] and several components in the mitogen-activated protein kinase (MAPK) pathway [44]. Therefore, we next examined whether miR-130a regulates c-Myc levels. As shown in Fig. 2A, overexpression of miR-130a significantly reduced the levels of both c-Myc protein and mRNA, compared to the negative mimic control, in U2OS cells. Conversely, suppression of endogenous miR-130a in U2OS cells by transfecting with miRIDIAN miR-130a hairpin inhibitor increased the levels of both c-Myc protein and mRNA as compared to the negative inhibitor control (Fig. 2C). Similar effects were also observed in primary human fibroblast WI38 cells (Figs. 2B and 2D), suggesting that the inhibition of c-Myc by miR-130a is not cell type-specific effect.

### miR-130a targets c-myc mRNA through the c-myc 3′-UTR

**Figure 3:**
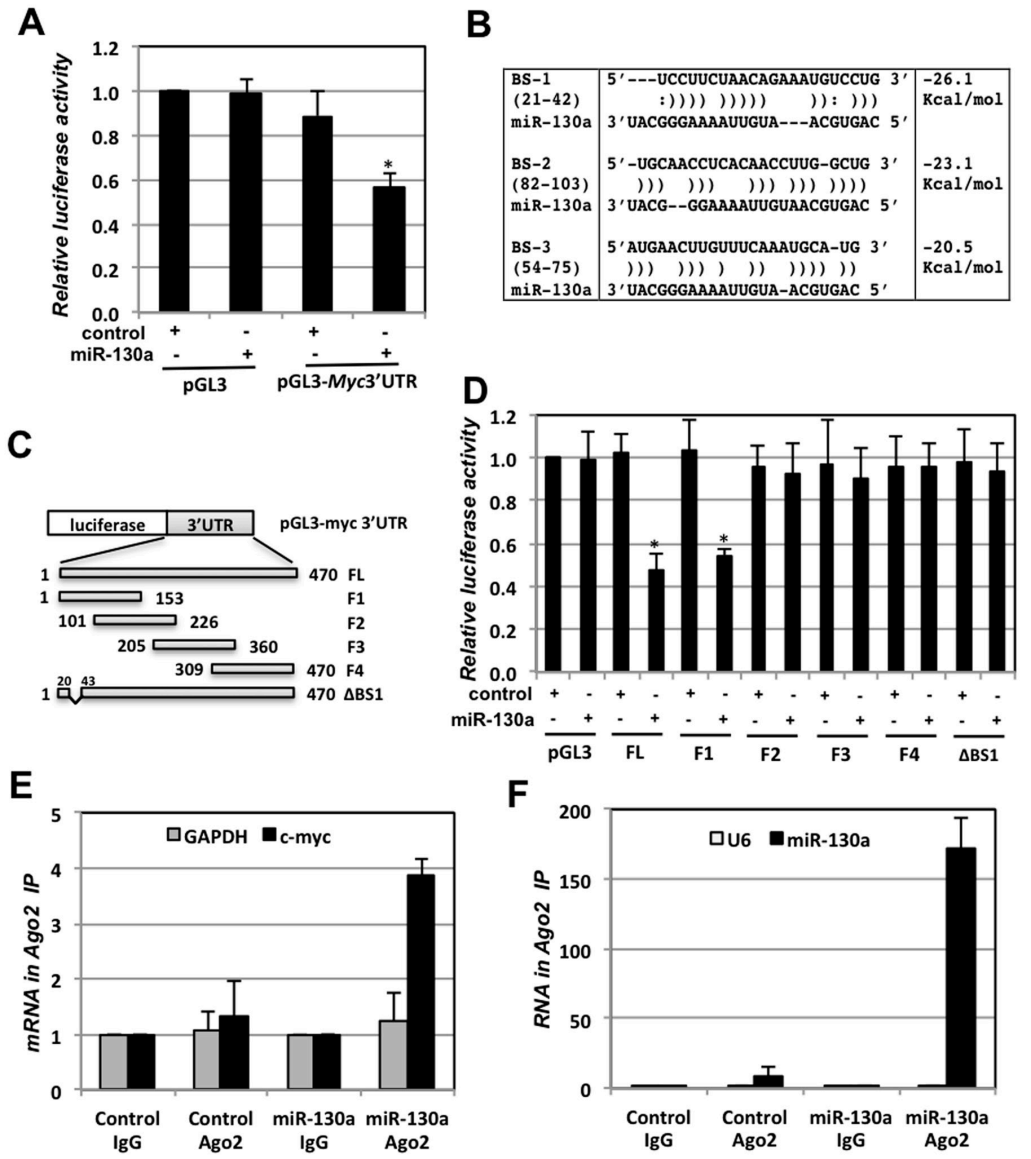
miR-130a targets *c-myc* mRNA through its 3′-UTR **(A)** Overexpression of miR-130a reduces the activity of luciferase reporter with *c-myc* 3′-UTR. 293 cells transfected with control pGL3 or pGL3-myc-3′UTR in the presence of β-gal plasmid together with control or miR-130a mimic as indicated were assayed for the relative luciferase activity normalized to β-gal expression. *p < 0.01, compared with cells transfected with pGL3-myc-3′UTR and control miRNA mimic. **(B)** Three putative miR-130a binding sites (BS-1, BS-2 and BS-3) in the *c-myc* 3′-UTR predicted by RNA22 program. **(C)** Schematic diagram of the pGL3-myc-3′UTR vectors. The first nucleotide after stop codon is indicated as “1”. **(D)** miR-130a regulates c-Myc via BS-1. 293 cells transfected with control or miR-130a mimic together with the indicated pGL3 or pGL3-myc-3′UTR vectors were assayed for the relative luciferase activity normalized to β-gal expression. *p < 0.01, compared with cells transfected with control miRNA mimic and corresponding luciferase reporters. **(E–F)** Ago2 associates with miR-130a at *c-myc* mRNA. U2OS cells transfected with control or miR-130a mimic were subjected to RNA-IP using control IgG or anti-Ago2 antibody, followed by RT-qPCR detection of *c-myc* and GAPDH mRNA (E) as well as U6 and miR-130a (F).

We next asked whether miR-130a directly targets *c-myc* mRNA at its 3′-UTR. 293 cells were co-transfected with control or miR-130a mimic together with control pGL3-promoter vector or pGL3-myc 3′UTR, which contains a full-length *c-myc* 3′-UTR at the 3′ end of *luciferase* mRNA, followed by measuring relative luciferase activity. As shown in Fig. 3A, overexpression of miR-130a significantly reduced the luciferase activity in cells transfected with pGL3-myc-3′UTR, but not the control pGL3 vector, suggesting that miR-130a targets *c-myc* mRNA through its 3′-UTR. We then searched for the potential miR-130a binding sites at the *c-myc* 3′-UTR. Although no conserved seed sequence for miR-130a binding was noted, analysis using RNA22 program as described [23], which allows seed mismatches [49], identifies three putative non-canonical “seedless” miR-130a binding sites (BS-1, BS-2, and BS-3) in the 5′ of the *c-myc* 3′-UTR with the miRNA:mRNA free folding energy cutoff –20 Kcal/mol (Fig. 3B). Therefore, we tested whether miR-130a targets *c-myc* mRNA at these sites using luciferase reporters containing different fragments of *c-myc* 3′-UTR (Fig. 3C). As shown in Fig. 3D, overexpression of miR-130a significantly reduced the luciferase activity in cells expressing pGL3-myc-3′UTR-FL or pGL3-myc-3′UTR-F1 plasmid containing the three putative miR-130a binding sites, whereas it did not significantly affect such activity in cells transfected with other pGL3 reporter containing *c-myc* 3′-UTR fragments lacking these sites (F2, F3 or F4) (Fig. 3D). Further, deletion of the first putative binding sites (BS-1, nt 21–42) (pGL3-myc-3′UTRΔBS1) with folding energy below the stringent cutoff (–25 Kcal/mol) [49] completely abolished the inhibition of luciferase activity upon miR-130a overexpression (Fig. 3D). Together, these results suggest that miR-130a targets *c-myc* mRNA through binding to the BS-1 site. To further confirm the miR-130a targeting of *c-myc* mRNA, we performed miR-130a transfection followed by RNA-IP using anti-Ago2 antibodies. As shown in Figs. 3E and 3F, both miR-130a and *c-myc* mRNA, but not U6 or *GAPDH* mRNA, were significantly enriched in the anti-Ago2, but not the control IgG, immunoprecipitates in cells transfected with miR-130a mimic. Thus, miR-130a directly targets *c-myc* mRNA at its 3′-UTR.

### Overexpression of miR-130a suppresses cell proliferation

**Figure 4:**
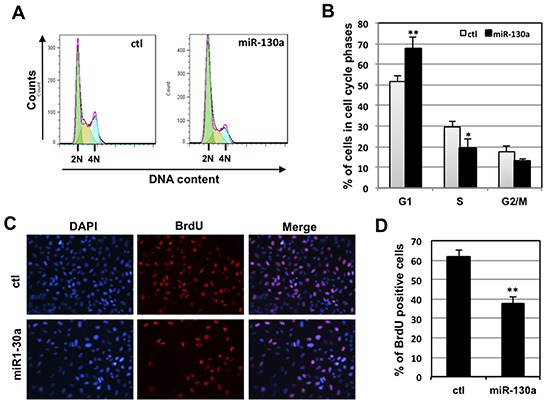
miR-130a inhibits cell proliferation **(A–B)** Overexpression of miR-130a inhibits cell cycle progression. U2OS cells were transfected with control or miR-130a mimic for 48 hours. The cells were trypsinized, stained with PI, and analyzed by flow cytometry. The histograms of PI staining from one representative experiment indicating the G1 (2N DNA), G2/M (4N DNA) and S (between G1 and G2/M phases) phases are shown in panel (A). The mean percentages of cells in different cell cycle phases from three independent experiments are shown in panel (B). *p < 0.05; **p < 0.01, compared with control transfected cells. **(C–D)** BrdU incorporation assays. U2OS cells were transfected with control or miR-130a mimic as above. At 48 hour post-transfection, the cells were incubated with BrdU for another 10 hours. The cells were fixed and stained with anti-BrdU antibodies (red) and DAPI (blue) (C). The average of BrdU-positive cells is shown in (D). **p < 0.01, compared with control transfected cells.

To understand the biological function of miR-130a inhibition of c-Myc, we examined whether miR-130a affects cell proliferation. To this end, U2OS cells were transfected with control or miR-130a mimic followed by cell cycle analysis. As shown in Fig 4A and 4B, overexpression of miR-130a significantly reduced the percentage of S phase cells with the concomitant accumulation of G1 phase cells, indicating the inhibition of cell cycle progression by miR-130a. BrdU incorporation assays (Figs. 4C, 4D) also showed significant reduction of S-phase cells by miR-130a transfection. These data clearly indicate that miR-130a negatively regulates cell cycle progression and proliferation.

### c-Myc is down-regulated following UV irradiation dependently on L11

**Figure 5:**
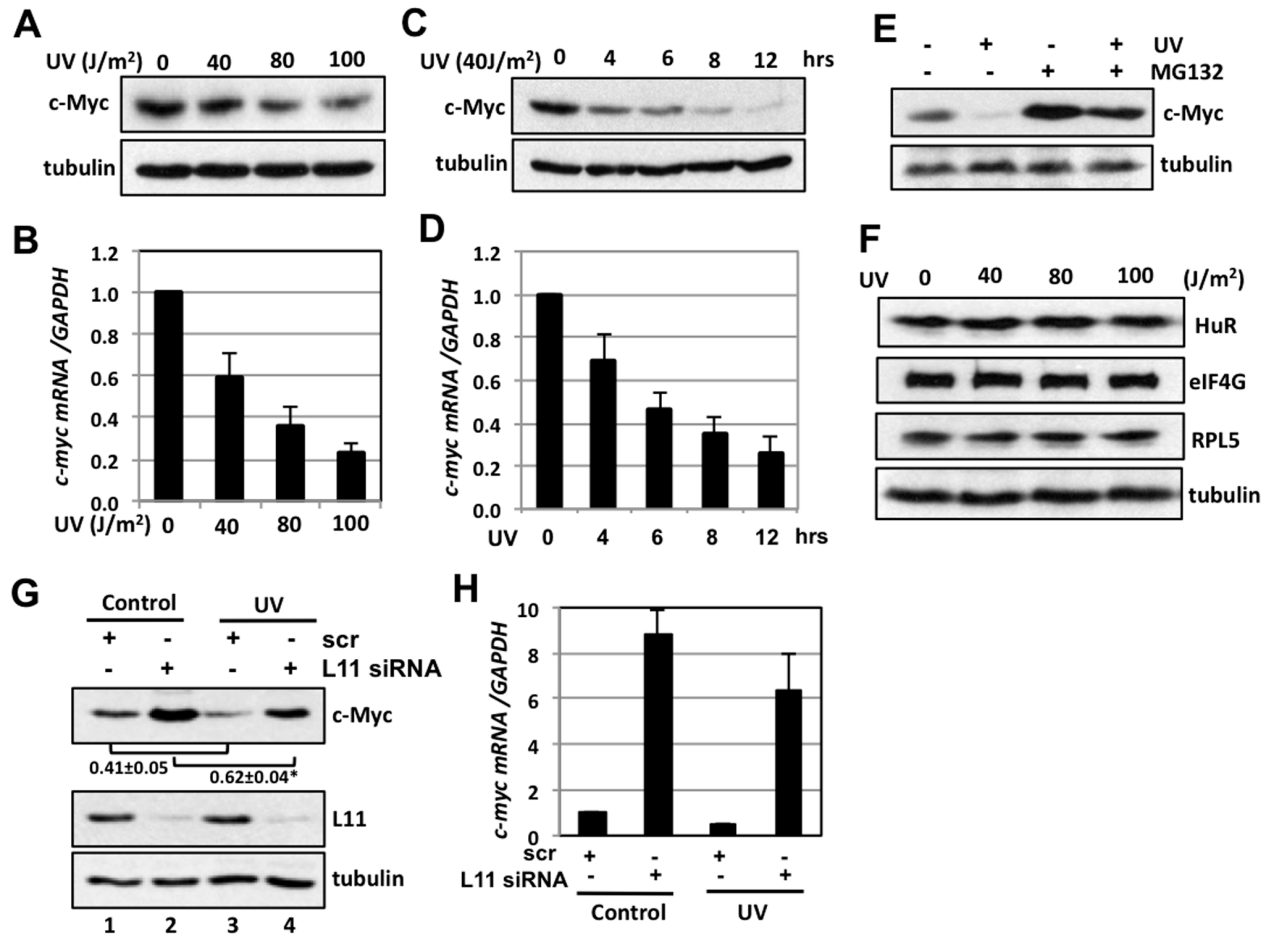
L11 is involved in UV-induced c-Myc downregulation **(A–D)** UV irradiation decreases c-Myc levels. U2OS cells were exposed to different dosages of UV (A) (B) or 40 J/m^2^ UV for different times (C) (D). The cells were assayed for the expression of c-Myc protein by IB (A) (C) and *c-myc* mRNA by RT-qPCR (B) (D). **(E)** MG132 treatment partially rescued the c-Myc reduction by UV treatment. U2OS cells treated with 40 J/m^2^ UV were cultured in the presence or absence of 40 μM MG132 for 6 hours followed by IB. **(F)** UV treatment does not reduce the levels of HuR, eIF4G and ribosomal protein L5 (RPL5). U2OS cells were treated with different dosages of UV for 6 hours and assayed by IB. **(G–H)** Knockdown of L11 abolished the c-Myc reduction by UV treatment. U2OS cells transfected with scrambled or L11 siRNA were treated with 40 J/m^2^ UV for 6 hours. The cells were subjected to IB detection of c-Myc protein (G) and RT-qPCR detection of *c-myc* mRNA (H). *p < 0.01, compared to scrambled RNA transfected cells.

To determine the physiological relevance of the L11-miR-130a regulation of c-Myc, we asked whether L11 recruits miR-130a to target c-Myc in response to stress. It has recently been shown that c-Myc protein is reduced by treatment of cells with UV irradiation [29] and DNA damaging agents [30], although the underlying mechanism is not completely understood. In agreement with these studies, we observed that c-Myc protein is reduced by UV treatment in U2OS cells (Fig. 5A and 5C). Interestingly, *c-myc* mRNA was also significantly reduced by UV treatment in a dose- and time-dependent manner (Figs. 5B and 5D), indicating that c-Myc is regulated at mRNA levels as well in response to UV-induced DNA damage. Also consistent with the previous study (30), UV-mediated c-Myc reduction was partially rescued by the treatment with the proteasome inhibitor MG132 (Fig. 5E). Thus, UV treatment leads to both degradation of existing c-Myc protein and the reduction of *c-myc* mRNA. Of note, the c-Myc reduction is not a general consequence of cellular response to UV irradiation, as the levels of several other tested proteins, including HuR, eIF4G, and ribosomal protein L5 (RPL5), were not decreased following UV treatment (Fig. 5F).

We have previously shown that the *c-myc* mRNA is down-regulated in response to ribosomal stress via the recruitment of miRISC to *c-myc* 3′-UTR by L11 [22]. Ribosomal stress is characterized by the disruption of the nucleolus, resulting in the relocation of the nucleolar components including ribosomal proteins (e.g. L11) into the nucleoplasm and the cytoplasm [40, 41]. Intriguingly, disruption of the nucleolus also occurs in cells following DNA damage, including UV irradiation [42]. Thus, we tested whether L11 may be involved in c-Myc down-regulation following UV treatment. U2OS cells transfected with scrambled or L11 siRNA were exposed to UV irradiation. As shown in Figs. 5G and 5H, knockdown of L11 partially abolished the reduction of both c-Myc protein (compare the ratio of lane 4 to lane 2 with the ratio of lane 3 to lane 1 in Fig. 5G, *p* < 0.01) and mRNA (Fig. 5H) caused by UV treatment, indicating that L11 plays a role in c-Myc down-regulation in response to UV irradiation.

### L11 promotes the recruitment of miR-130a to *c-myc* mRNA in response to UV treatment

**Figure 6:**
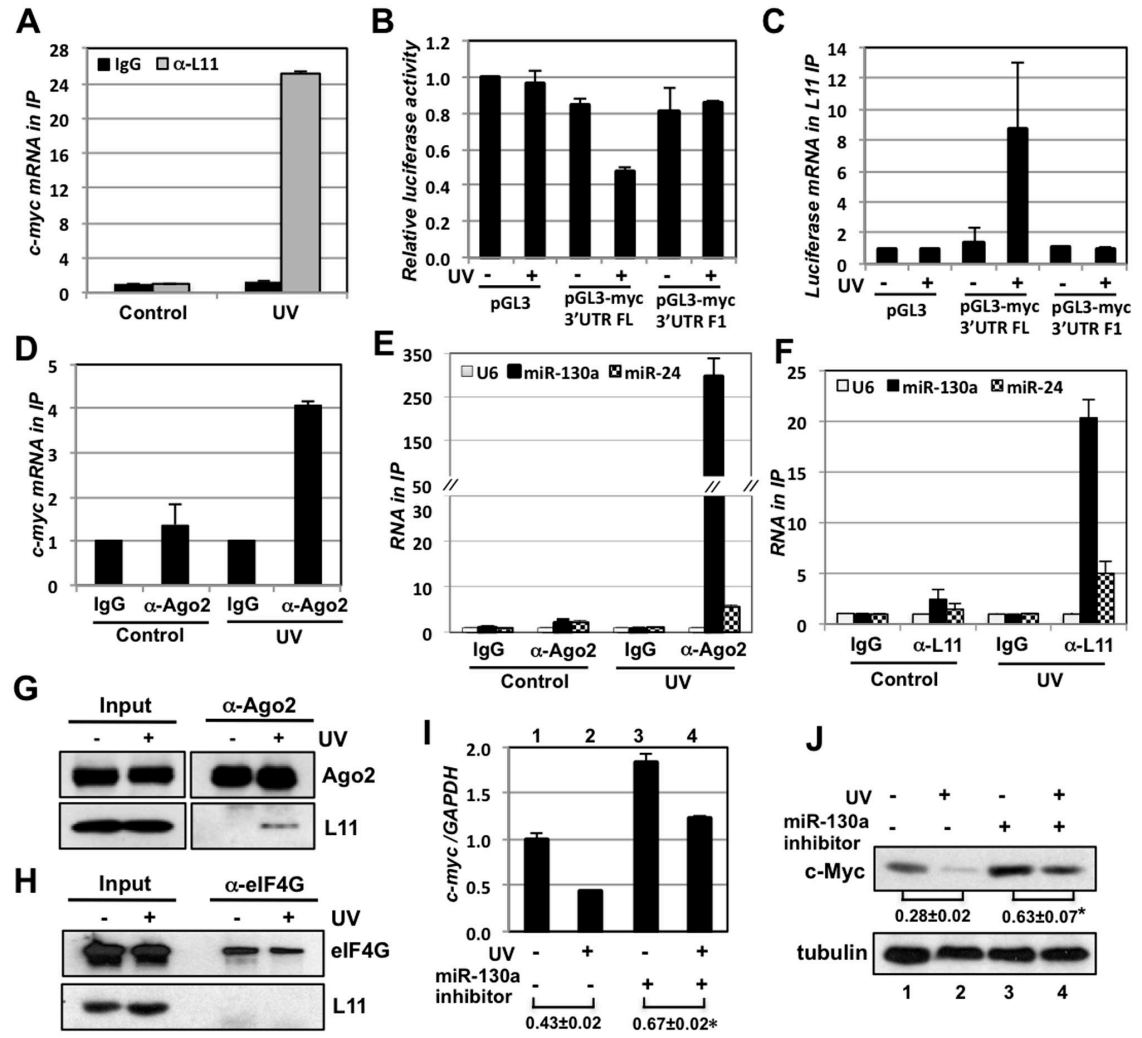
L11 recruits miR-130a-loaded miRISC to *c-myc* mRNA in response to UV irradiation **(A)** UV treatment increases the L11 binding to *c-myc* mRNA. U2OS cells treated without or with UV were subjected to RNA-IP using control IgG or anti-L11 antibodies, followed by RT-qPCR assays. **(B–C)** L11 inhibition of c-Myc in response to UV requires its binding to the *c-myc* 3′-UTR. 293 cells transfected with pGL3, pGL3-myc 3′-UTR-FL, or pGL3-myc 3′-UTR-F1 were treated with or without UV. The cells were then assayed for the relative luciferase activity normalized to β-gal expression (B) and subjected to RNA-IP using anti-L11 antibodies, followed by RT-qPCR detection of the *luciferase* mRNA. **(D)** UV treatment increases Ago2 binding to *c-myc* mRNA. U2OS cells treated with or without UV were subjected to RNA-IP using control IgG or anti-Ago2 antibodies, followed by RT-qPCR detection of *c-myc* mRNA (D). **(E–F)** UV treatment increases the binding of L11 and Ago2 to miR-130a, and, to a less extent, to miR-24. U2OS cells treated with or without UV were subjected to RNA-IP using control IgG or anti-Ago2 (E) or anti-L11 (F) antibodies, followed by RT-qPCR detection of miR-130a, miR-24 and the control U6 RNA. **(G–H)** UV treatment increases the L11 binding to Ago2, but not eIF4G. U2OS cells treated with or without UV were subjected to co-IP with anti-Ago2 (G) and anti-eIF4G (H) antibodies followed by IB. **(I–J)** Inhibiting miR-130a abolishes c-Myc reduction by UV treatment. U2OS cells transfected with control or miR-130a inhibitor were treated with or without UV. The cells were assayed for the expression of *c-myc* mRNA by RT-qPCR (I) and c-Myc protein by IB (J). *p < 0.05, compared the ratio of lane 4 to lane 3 with the ratio of lane 2 to lane 1. In all above assays, cells were treated with 40 J/m^2^ UV and harvested at 6 hours post-treatment.

We then examined whether L11 promotes the recruitment of the miR-130a-loaded miRISC to *c-myc* mRNA in response to UV treatment. First, U2OS cells treated with or without UV were subjected to RNA-IP using control IgG or anti-L11 antibodies, followed by RT-qPCR assays. As shown in Fig. 6A, L11 binding to *c-myc* mRNA was drastically increased in cells treated with UV compared to the control cells, confirming that UV treatment promotes the L11 binding to *c-myc* mRNA. Our previous study showed that L11 binds to the 3′-end (nt 361 to 470) of the *c-myc* 3′-UTR [22]. To verify that UV treatment promotes L11 binding to the *c-myc* 3′-UTR, we transfected 293 cells with pGL3, pGL3-myc-3′UTR-FL, or pGL3-myc-3′UTR-F1 plasmid as diagramed in Fig. 3C (the F1 fragment contains the miR-130a binding site, but lacks the L11-binding site), followed by UV treatment. As shown in Fig. 6B, UV treatment significantly reduced the luciferase activity in cells expressing pGL3-myc-3′UTR-FL, but not the control pGL3 or pGL3-myc-3′UTR-F1 plasmid lacking the L11 binding site. Consistently, UV treatment significantly increased the binding of L11 to the luciferase mRNA in cells transfected with pGL3-myc 3′-UTR, but not the control pGL3 or pGL3-myc-3′UTR-F1 (Fig. 6C). These data reveal that UV treatment increases the L11 binding to the *c-myc* 3′-UTR and inhibits c-Myc expression.

To examine whether L11 promotes the recruitment of miR-130a-loaded miRISC to *c-myc* mRNA in response to UV, U2OS cells treated with or without UV were subjected to IP with anti-Ago2, anti-L11 antibodies, or control IgG. As shown in Figs. 6D and 6E, the binding of Ago2 to both *c-myc* mRNA and miR-130a was markedly increased in the cells treated with UV compared to the controls. L11 binding to miR-130a was also drastically increased in cells treated with UV (Fig. 6F). Interestingly, although L11 recruits miR-24 to the *c-myc* 3′-UTR in response to ribosomal stress (22), the binding of L11 and Ago2 to miR-24 following UV treatment was much less robust compared to that of miR-130a (Figs. 6E and 6F), suggesting that miR-130a plays a prevalent role over miR-24 in c-Myc down-regulation in response to UV irradiation. In addition, co-IP analysis showed that the interaction between L11 and Ago2 was increased in both U2OS (Fig. 6G) and 293 cells (data not shown) by UV treatment. This interaction is specific, as we did not detect the interaction between L11 and the eIF4G, an essential scaffold protein in the translation initiation eIF4E complex that allows ribosome binding to the 5′-cap of mRNAs during an early step in the initiation of translation (59) and may also play a role in miRNA-mediated translation inhibition (60), in cells either treated with or without UV (Fig. 6H). Together, these results demonstrate that L11 promotes the recruitment of miR-130a-loaded miRISC to *c-myc* mRNA and down-regulates *c-myc* mRNA in response to UV irradiation.

Finally, we found that inhibiting miR-130a by the miR-130a inhibitor significantly abolished UV-induced reduction of c-Myc mRNA (Fig. 6I, compare the ratio of column 4 to column 3 with the ratio of column 2 to column 1) and protein (Fig. 6J, compare the ratio of lane 4 to lane 3 with the ratio of lane 2 to lane 1). Thus, these results demonstrate that L11 plays an important role in c-Myc down-regulation in response to UV irradiation by promoting miR-130a-loaded miRISC to *c-myc* mRNA.

### L11 recruits miR-130a to target c-myc mRNA in the cytoplasm

**Figure 7:**
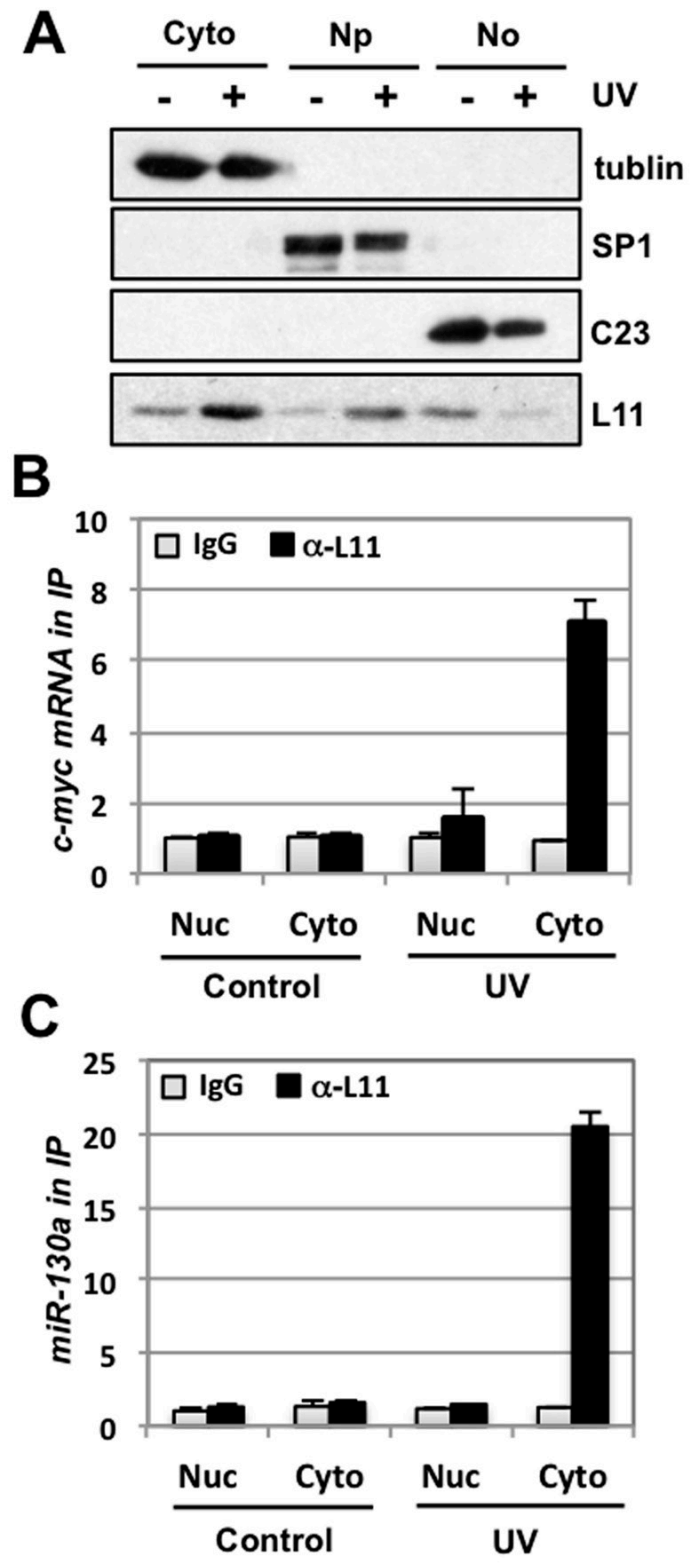
UV irradiation promotes L11 interaction with miR-130a and *c-myc* mRNA in the cytoplasm **(A)** UV treatment releases L11 from the nucleolus into the nucleoplasm and the cytoplasm. U2OS cells treated with or without 40 J/m^2^ UV for 6 hours were subjected to isolation of cytoplasm (Cyto), nucleoplasm (Np), and the nucleolus (No) fractions, followed by IB detection of indicated proteins. Tubulin, SP1, and nucleolin (C23) were used as cytoplasm, nucleoplasm and nucleolar markers, respectively. **(B–C)** UV treatment increases L11 binding to miR-130a and *c-myc* mRNA in the cytoplasm. The cytoplasmic and the nuclear (Nuc) fractions isolated from U2OS cells treated with or without 40 J/m^2^ UV for 6 hours were immunoprecipitated with anti-L11 antibodies or control rabbit IgG, followed by RT-qPCR detection of *c-myc* mRNA (B) and miR-130a (C).

To test how L11 targets *c-myc* mRNA in response to UV treatment, we examined whether UV treatment could increase L11 levels in the cytoplasm. U2OS cells treated with or without UV were fractionated into the cytoplasm, nucleoplasm and nucleolar fractions, followed by IB. As shown in Fig. 7A, UV treatment significantly increased the levels of L11 in both the nucleoplasm and the cytoplasm, whereas the nucleolar L11 was reduced by UV treatment. This is consistent with previous report showing that UV damage causes nucleolar disruption [42]. RNA-IP assays using the cytoplasmic and nucleoplasmic lysates with control or anti-L11 antibodies showed that UV treatment significantly increased the L11 binding to both *c-myc* mRNA (Fig. 7B) and miR-130a (Fig. 7C) in the cytoplasm, but not in the nucleoplasm. These results indicate that in response to UV damage, L11 is released form the nucleolus to the cytoplasm where it recruits miR-130a/miRISC to the *c-myc* 3′-UTR, leading to *c-myc* mRNA decay.

## DISCUSSION

Herein, we have identified that miR-130a is a novel L11-associated miRNA that suppresses c-Myc expression. Several lines of evidence demonstrate that miR-130a directly targets *c-myc* mRNA: (1) Overexpression of miR-130a reduced the levels of c-Myc protein and mRNA, whereas inhibiting endogenous miR-130a significantly increased the levels of c-Myc protein and mRNA (Fig. 2); (2) miR-130a expression reduced the luciferase reporter activity in cells transfected with pGL3-*c-myc* 3′-UTR, but not the control pGL3 plasmid (Fig. 3A). Further mapping analysis with pGL3-*c-myc* 3′-UTR mutants showed that miR-130a binds to the 5′ end BS-1 region (nt 21-42) in the *c-myc* 3′-UTR (Fig. 3D); (3) RNA-IP assays showed that *c-myc* mRNA was enriched in anti-Ago2 immunoprecipitates when miR-130a is overexpressed in cells (Fig. 3E). Functionally, we show that overexpression of miR-130a significantly inhibited cell cycle progression and suppressed cell proliferation (Fig. 4). These results indicate that miR-130a can suppress cell proliferation via targeting *c-myc* mRNA.

miR-130a has recently emerged as a key miRNA that inhibits cancer cell proliferation, invasion and migration by targeting other cellular proteins that promote cell proliferation or have oncogenic potential. For example, miR-130a targets MET receptor tyrosine kinase to suppress lung cancer cell migration and promote TRAIL-induced apoptosis [43]. miR-130a, jointly with miR-203 and miR-205, targets several components in the MAPK and androgen receptor (AR) pathways to induce apoptosis and cell cycle arrest in prostate carcinoma cells [44]. miR-130a also targets ATG2B and DICER1 to inhibit autophagy and trigger killing of chronic lymphocytic leukemia cells (50). Consistently, miR-130a has been shown down-regulated in multiple cancers [43, 44, 50] and leukemias [51], Together with our observation that miR-130a directly targets c-Myc, these results reveal that miR-130a may possess tumor suppressor function.

Interestingly, we found that L11 promotes the recruitment of miR-130a to *c-myc* mRNA to suppress c-Myc expression in response to UV irradiation. UV has been shown to induce c-Myc protein degradation and it was thought that c-Myc down-regulation is part of the global cell response to DNA damage, complementary to the activation of p53 to stall cell cycle progression, thereby preventing genomic instability [29]. Our results here showed that UV also causes *c-myc* mRNA decay through an L11- miR-130a-mediated mechanism. UV treatment significantly increased the binding of L11 and Ago2 to miR-130a and *c-myc* mRNA as well as the interaction between L11 and Ago2 (Fig. 6). Knockdown of L11 (Figs. 5G and 5H) or inhibiting miR-130a (Figs. 6I and 6J) significantly blocked UV treatment-induced c-Myc reduction. These results reveal that upon UV treatment, L11 promotes miR-130a-loaded miRISC to target *c-myc* mRNA. Our finding is additionally supported by a recent study showing that UV triggers a cell-cycle-dependent relocalization of Ago2 into stress granules and various miRNA-expression changes, which mediate gene regulation earlier than most transcriptional responses [52]. Thus, c-Myc down-regulation via miRNA-mediated mRNA decay is a specific cellular response to UV irradiation, rather than a general cellular stress outcome.

Our study also suggests that L11 acts as a stress-induced accessory factor to facilitate Ago2-miR-130a loading onto *c-myc* mRNA. Under normal condition, miR-130a weakly associates with Ago2 and the basal level of *c-myc* mRNA targeting by Ago2 is minimal (Fig. 6), likely due to the lack of significant amount of free L11 or proper modification of L11. Upon UV irradiation, L11 is relocalized from the nucleolus to the cytoplasm where it targets miR-130a to *c-myc* mRNA (Fig. 7). This is consistent with the notion that UV irradiation disrupts the nucleolus [42] and thus inducing ribosomal stress. It is interesting to examine whether posttranslational modification of L11 may contribute to this process. Of note, we have previously shown that L11 binds to the 3′-end of *c-myc* 3′-UTR [22] whereas miR-130a-binding site is located at the 5′-end of *c-myc* 3′-UTR (Fig. 3D), suggesting that structural accessibility contributes to this L11-recruited miR-130a-*c-myc* mRNA complex. The 5′ BS-1 (nt 21–42) region contains a loop structure (consistent with the recent finding that single stranded (loop) sites are more accessible for Ago2 binding and more likely to be true miRNA targets [53]), that is located close to the 3′ end in predicted secondary structure of *c-myc* 3′-UTR (not shown), suggesting that the secondary structure of the *c-myc* 3′-UTR is accessible to the L11-miR-130a-Ago2 complex. Alternatively, L11 binding may change the *c-myc* 3′UTR conformation, allowing the targeting by miR-130a/miRISC. Nevertheless, our results strongly suggest that upon UV irradiation, L11 recruits miR-130a-loaded miRISC to target *c-myc* 3′-UTR, leading to *c-myc* mRNA decay, demonstrating a novel mechanism underlying c-Myc downregulation in response to UV-induced DNA damage.

## EXPERIMENTAL PROCEDURES

### Cell culture and UV irradiation

Human embryonic kidney epithelial 293 cells and human osteosarcoma U2OS cells were cultured in Dulbecco's modified Eagle's medium (DMEM) supplemented with 10% fetal bovine serum (FBS), 50 U/ml penicillin and 0.1 mg/ml streptomycin at 37°C in a 5% CO_2_ humidified atmosphere. Human diploid lung fibroblast WI38 cells were cultured in DMEM supplemented with 15% FBS and MEM nonessential amino acids (Gibco) [22, 54]. Cells were irradiated with UV-C at 50%–70% confluency in the absence of medium without the lid as described previously [55]. After UV irradiation, the medium was added to the plates.

### Plasmids and antibodies

The Flag-tagged L11 (Flag-L11) and pGL3-myc 3′UTR luciferase reporter plasmids were described previously [22], except that the mutant with the deletion of the BS-1 (pGL3-myc-3′UTRΔBS1) was constructed by PCR using pGL3-myc 3′UTR plasmid as a template. The primers used are: 5′- CGCTCTAGAGGAAAAGTAAGGAAAACGATAGCAA TCACCTATGAACTTG-3′ (forward) and 5′-CGCTCTAGA TTGGCTCAATGATATATTTGCCA G-3′ (reverse). The PCR product was then cloned into pGL3-promoter plasmid (Promega) at the Xba I site and sequenced. Anti-Flag (M2; Sigma), rabbit polyclonal anti-Ago2 (Millipore), mouse monoclonal anti-Ago2 (Abcam), and mouse polyclonal anti-Myc (Y69; Abcam) antibodies were purchased. Rabbit polyclonal anti-L11 antibodies were previously described [56].

### Transfection, immunoblot, and co-immunoprecipitation analyses

Cells were transfected with plasmids using TransIT-LT1 (Mirus Bio Corporation, for U2OS cells), TransFectin (Bio-Rad, for 293 cells), or Lipofectamine® 2000 (Invitrogen, for WI38 cells) reagents following the manufacturers' protocols. Cells were harvested at 48 hours posttransfection and lysed in lysis buffer consisting of 50 mM Tris-HCl (pH 8.0), 0.5% Nonidet P-40, 1 mM EDTA, 150 mM NaCl, 1 mM phenylmethylsulfonyl fluoride (PMSF), 1 mM dithiothreitol (DTT), 1 μg/ml pepstatin A, and 1 mM leupeptin. Equal amounts of cell lysates were used for immunoblot (IB) analysis as described previously (54). Co-immunoprecipitation (co-IP) was conducted as described previously [22, 54].

### Immunoprecipitation of protein-associated RNAs (RNA IP)

Immunoprecipitation of RNA-protein complexes was performed as described [22]. Briefly, cells were lysed in polysome lysis buffer (100 mM KCl, 5 mM MgCl_2_, 10 mM HEPES [pH 7.0], 0.5% Nonidet P-40, 1 mM DTT, 100 U/ml RNase inhibitor) supplemented with 20 mM EDTA and protease inhibitors on ice for 20 minutes. After centrifugation, the supernatants were pre-cleared with protein A-Sepharose beads and then diluted (1:10 [vol/vol]) in NT2 buffer (50 mM Tris [pH 7.4], 150 mM NaCl, 1 mM MgCl_2_, 0.05% Nonidet P-40, 1 mM DTT, 100 U/ml of RNase inhibitor) supplemented with 20 mM EDTA and protease inhibitors and incubated with primary antibodies at 4°C for 4 hours, followed by incubation with protein A/G beads for an additional 2 hours. The beads were washed five times with NT2 buffer supplemented with protease inhibitors. The bead-bound protein-RNA complexes were then treated with DNase I and proteinase K and eluted twice with NT2 buffer containing 0.1% SDS. RNAs were extracted from the elution with phenol-chloroform and ethanol precipitation and subjected to RT-qPCR assays.

### Luciferase reporter assays

Cells were transfected with pCMV-β-galactoside (β-gal) and luciferase reporter plasmid pGL3, pGL3-myc-3′UTR or its mutants, together with control or miR-130a mimic. Luciferase activity was determined and normalized by calculating β-gal activity as described previously [22].

### RT-qPCR analysis

Total RNA was isolated from cells using TRIzol reagent (Invitrogen) or Qiagen miRNeasy mini Kit (Qiagen, Valencia, CA). Reverse transcriptions were performed as described [22, 57]. qPCR was performed using an ABI StepOne real-time PCR system (Applied Biosystems) and iTaqTM Universal SYBR Green Supermix (Bio-Rad) for mRNA expression determinations as described previously [22, 57]. Analysis of mature miRNAs expression was performed using a TaqMan miRNA assay kit (Applied Biosystems) following the manufacturer's protocol [22]. All reactions were carried out in triplicate. Relative gene expression levels were calculated using the ΔCτ method following the manufacturer's instructions. The primers for *c-Myc*, *luciferase* and *GAPDH* were previously described [22].

### RNA interference (RNAi) and miRNA transfection

The 21-nt siRNA duplexes with a 3′ dTdT overhang were synthesized by Dharmacon Inc. (Lafayette, CO). The target sequences for L11 and control scramble II RNA were previously described [22]. The miRIDIAN miR-130a mimic, negative control cel-miR-67 mimic, miRIDIAN miR-130a hairpin inhibitor and miRIDIAN microRNA inhibitor negative control were purchased from Dharmacon Inc. These siRNA duplexes (100 nM) and miRNA mimics/inhibitors (25 to 50 nM) were introduced into cells using SilentFect lipid reagent (Bio-Rad) following the manufacturer's protocol. The cells were analyzed 48 hours after transfection.

### Cell cycle analysis

Cells were harvested, washed with PBS buffer and stained with propidium iodide (PI; Sigma) staining buffer (50 μg/ml PI, 200 μg/ml RNase A, and 0.1% Triton X-100 in PBS) at 37°C for 30 min. The cells were measured for DNA content using a Becton Dickinson FACScan flow cytometer. Data were analyzed using FlowJo software program.

### Bromodeoxyuridine (BrdU) incorporation assay

BrdU incorporation assays were conducted as described previously [56, 58]. Briefly, cells were labeled with 10 μM BrdU for 10 hours and then fixed with 95% ethanol and 5% acetic acid and treated with 2M HCl containing 1% Triton X-100. The cells were stained with monoclonal anti-BrdU antibody (Roche), followed by staining with Alexa Fluor 546 (red) goat anti-mouse antibodies and 4′, 6′-diamidino-2-phenylindole (DAPI). Stained cells were imaged in five randomly selected fields with an EVOS fluorescence microscopy. The BrdU-positive cells were counted and quantified using the ImageJ software.

### Cell fractionations

Cytoplasmic and nuclear fractions were isolated from cells as previously described [22]. To isolate nucleolar fraction, the nuclear pellets were resuspended in buffer S1 containing 0.25 M sucrose and 10 mM MgCl_2_, layered over buffer S2 containing 0.35 M sucrose and 0.5 mM MgCl_2_, and centrifuged at 1,430g for 10 min at 4°C. The pelleted nuclei were resuspended in buffer S2 followed by sonication. The sonicated nuclei were then layered over buffer S3 containing 0.88 M sucrose and 0.5 mM MgCl_2_ and centrifuged at 3,000g for 10 min at 4°C. The pellet contained purified nucleoli, and the supernatant represented the nucleoplasm [22].

### Statistical analysis

All the statistical differences were analyzed by Student's *t*-test. *p* < 0.05 was considered statistically significant.
